# Asparagine Synthetase Deficiency: Neuropathological Evidence of Disrupted Cortical Development

**DOI:** 10.1002/jmd2.70126

**Published:** 2026-09-13

**Authors:** Mihaela Bobić, Goran Sedmak, Ema Bokulić, Tila Medenica, Tamara Žigman, Bernarda Medlobi Vinković, Viktorija Antolović, Nenad Kešin, Ivan Lehman, Ana Škaričić, Mirjana Novoselec, Josipa Mateševac, Monica Kirigin Kalamar, Sanda Huljev Frković, Ivo Barić, Danijela Petković Ramadža

**Affiliations:** ^1^ Croatian Institute for Brain Research University of Zagreb, School of Medicine Zagreb Croatia; ^2^ Scientific Centre of Excellence for Basic, Clinical and Translational Neuroscience University of Zagreb, School of Medicine Zagreb Croatia; ^3^ Department of Biology University of Zagreb, School of Medicine Zagreb Croatia; ^4^ Department of Pediatrics University Hospital Centre Zagreb Zagreb Croatia; ^5^ Department of Pediatrics University of Zagreb, School of Medicine Zagreb Croatia; ^6^ Neonatology Unit County Hospital Čakovec Čakovec Croatia; ^7^ Special Hospital for Chronic Diseases in Children Gornja Bistra Croatia; ^8^ Department of Laboratory Diagnostics University Hospital Centre Zagreb Zagreb Croatia; ^9^ Department of Diagnostic and Interventional Neuroradiology University Hospital Centre Zagreb Zagreb Croatia; ^10^ Department for Functional Genomics, Centre for Translational and Clinical Research University Hospital Centre Zagreb Zagreb Croatia; ^11^ Clinical Department of Pathology and Cytology “Ljudevit Jurak” University Hospital Center “Sestre Milosrdnice” Zagreb Croatia

**Keywords:** *ASNS* gene, asparagine, epilepsy, microcephaly, neuronal migration

## Abstract

Asparagine synthetase deficiency (ASNSD) is a rare metabolic disease causing congenital microcephaly, severe developmental delay, and spastic quadriplegia. Although the central nervous system is severely affected, other organ systems appear unaffected by asparagine deficiency. We present an infant homozygous for the mutation c.904‐1G>A in the *ASNS* gene, whose clinical presentation and radiological findings were typical for ASNSD. Following the patient's death at the age of 6 months, histological and immunohistochemical examination of the telencephalon revealed a vast disturbance of migration of neuronal subpopulations, consequently severe disorganization of cortical layers, and thinning of the cerebral cortex. These findings provide novel insights into disease pathogenesis and may explain the hallmark features of ASNSD, including microcephaly and epilepsy.

## Synopsis

Impaired neuronal migration is an important pathogenic mechanism underlying microcephaly and neurological dysfunction in asparagine synthetase deficiency.

## 1 Introduction

Asparagine synthetase deficiency (ASNSD) (OMIM #615574, ORPHA 391376) is a rare neurometabolic disease caused by biallelic pathogenic variants in the *ASNS* gene [1, 2]. Human asparagine synthetase (ASNS, EC 6.3.5.4) is a cytoplasmic enzyme that catalyzes the ATP‐dependent conversion of aspartate to asparagine using glutamine as a nitrogen donor [3]. ASNSD clinical features include congenital microcephaly, severe developmental delay, and axial hypotonia followed by spastic quadriplegia [1, 2]. Additional symptoms comprise apnea, irritability, seizures, cortical blindness, and feeding difficulties (gastroesophageal reflux, vomiting, and swallowing dysfunction) [1, 4, 5]. ASNSD is associated with high mortality, with half of affected children not surviving beyond their first year [6, 7, 8]. The diagnosis may be established through cerebrospinal fluid (CSF) amino acid analysis, which typically reveals reduced asparagine concentration, whereas plasma asparagine is decreased in about 50% of affected patients [2, 9]. However, as some ASNSD patients may have normal amino acid concentrations, genetic testing is superior to biochemical analysis for diagnosing this rare neurometabolic disorder [10].

The pathophysiology of this severe neurological phenotype remains poorly understood. Nevertheless, asparagine appears to play a critical role in fetal brain development, as its deficiency results in congenital microcephaly and profound neurological dysfunction, while other organ systems remain unaffected [11, 12, 13, 14]. Neuroradiological assessment typically shows reduced cortical gyration, generalized cerebral atrophy, and cerebellar vermis hypoplasia, whereas the underlying histopathological mechanisms have yet to be elucidated [2, 12, 15, 16].

In this report, we describe the clinical, biochemical, and neuroradiological features of an infant with ASNSD, along with the corresponding brain histopathological findings. Written informed consent for publication of clinical data, imaging findings, and postmortem analyses was obtained from the patient's parent.

## 2 Methods

### 2.1 Tissue Samples

The brain of the deceased patient, and two postmortem control brains of male infants that died at the ages of 3 and 7 months from the Zagreb Neuroembryological Collection, were analyzed in the histological/immunohistochemical part of the study [17]. The latter brain specimens were obtained from autopsies of the infants who died from sudden infant death syndrome. These brains were considered normal based on clinical and histopathological assessment. The study was approved by the Ethics Committee of the University of Zagreb School of Medicine in accordance with the Declaration of Helsinki (all analyzed brain samples are part of the Zagreb Neuroembryological Collection and were collected with the prior approval of the Ethical Committee of the University of Zagreb School of Medicine (251‐59‐10106‐23‐111/152) and with the approval of the next of kin or legal guardian). According to the institutional policy, separate ethical approval was not required for the publication of this single case report.

### 2.2 Histology and Immunohistochemistry

The entire brains were fixed by immersion in 4% formaldehyde in 0.1 M phosphate‐buffered saline (PBS, pH = 7.4) and cut into tissue blocks, which were further embedded in paraffin, cut in the coronal plane to 10 μm thick sections, and stained by cresyl‐violet (Nissl) staining to delineate histoarchitectonic transient developmental compartments. Histopathological and immunohistochemical analyses were restricted to the cerebral hemispheres; the brainstem and cerebellum were not examined. Neighboring sections were processed immunohistochemically as follows: after dewaxing through xylol immersion and subsequent rehydration in a graded series of ethanol solutions, sections were washed in PBS. For the heat‐induced antigen retrieval, sections were immersed in a citrate buffer (pH = 6.0) and heated in a microwave, then cooled at room temperature (RT) and washed again in PBS. The sections were pretreated in 0.3% H_2_O_2_ in a 3:1 mixture of methanol and redistilled water, washed in PBS, and immersed for 2 h in a blocking solution containing 5% bovine serum albumin and 0.5% Triton X‐100 in PBS (all from Sigma, St. Louis, MO, USA) at RT. Incubation in primary antibodies was done for 24 h at 4°C for the antibodies: (1) anti‐NeuN (rabbit, 1:1000), Abcam (ab104225, RRID: AB_10711153); (2) anti‐PARV (rabbit, 1:3000), Abcam (ab11427, RRID: AB_298032); (3) anti‐CALR (mouse, 1:5000), Swant (6B3, RRID: AB_10000320); (4) anti‐GFAP (rabbit, 1:1000), Agilent (Z0334, RRID: AB_10013382); (5) anti‐CALB, (rabbit, 1:2000), Swant (CB38, RRID:AB_10000340); (6) anti‐SMI‐99 (anti‐MBP), (mouse, 1:500), BioLegend (808 401, RRID:AB_2564741).

Sections were further incubated with appropriate secondary antibodies in the diluted blocking solution (1:200) for 1 h at RT (Vectastain ABC kit, Vector Laboratories, Burlingame, USA), followed by the Vectastain ABC reagent (streptavidin‐peroxidase complex) for 1 h at RT, and rinsed in PBS. Peroxidase activity was visualized with Ni‐3,3‐diaminobenzidine (Sigma D0426, St. Louis, MO, USA). After drying at RT, sections were cleared in BioClear (BioGnost) and covered with BioMount (BioGnost). Negative controls were included in all immunohistochemical experiments by replacing the primary antibody with the blocking solution.

### 2.3 Imaging

Qualitative analysis of the stained histological sections was performed using an Olympus BX53 light microscope, and images were captured with an Olympus UC‐90 digital camera (Olympus Corporation, Shinjuku, Tokyo, Japan) or using a high‐resolution digital slide scanner Nano‐Zoomer 2.0RS (Hamamatsu, Japan).

## 3 Case Report

**FIGURE 1:**
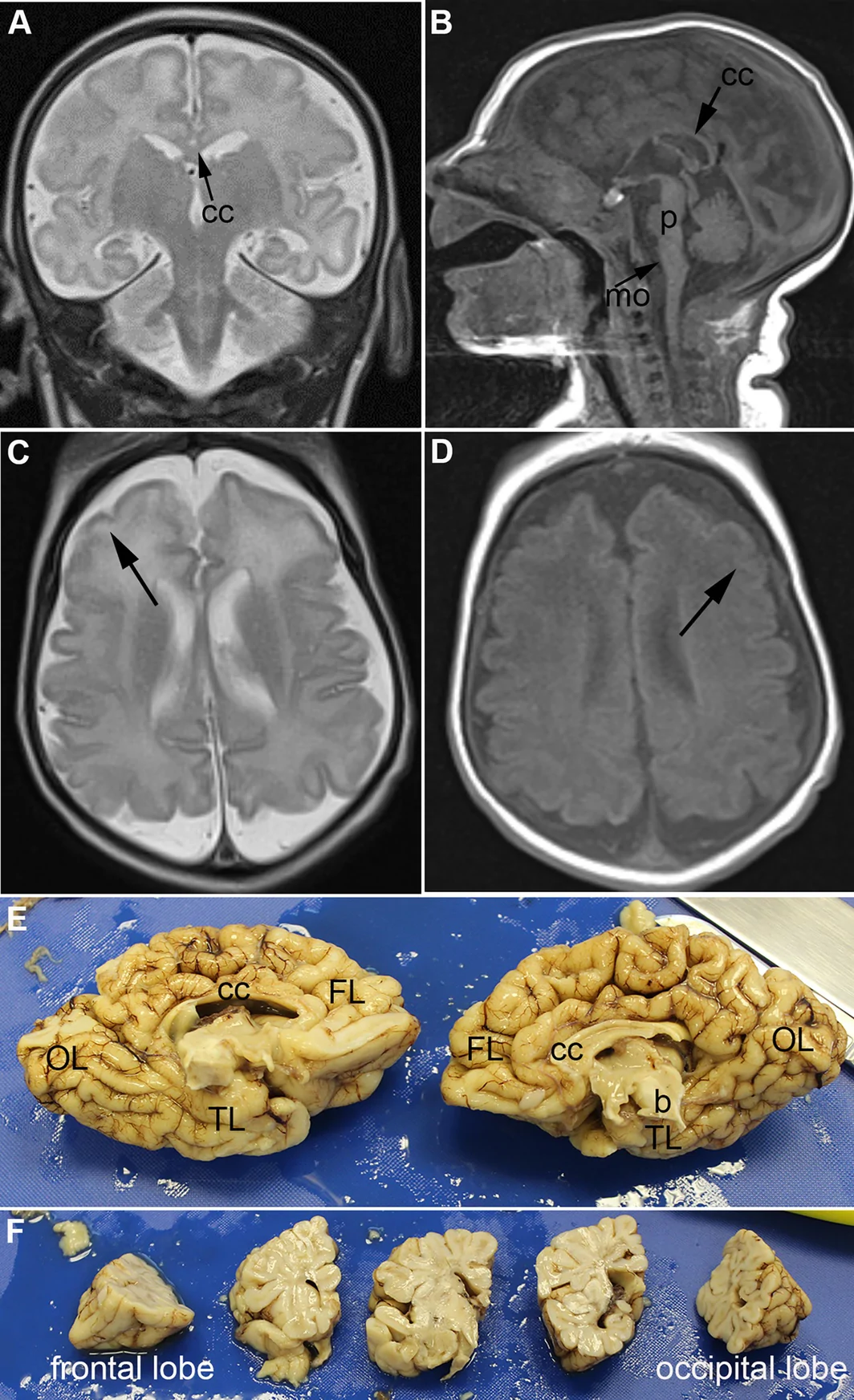
MRI in the second week of life and postmortem macroscopic photographs (6 months of age). MRI taken in the second week of life showed a simplified tertiary gyrification pattern (A, arrows in C and D), a thinner corpus callosum (A, B, also visible in E) than expected, and hypoplastic medulla oblongata and pons (B). The ventricular system was normally positioned and of normal size, without evidence of hydrocephalus (A–D). The medial view of the telencephalic hemispheres is shown in (E), while coronal slices are displayed in (F). MRI sequences: (A) T2 coronal plane, (B) T1 mid‐sagittal plane, (C) T2 axial plane, (D) T1 axial plane. b, brainstem; CC, corpus callosum; FL, frontal lobe; mo, medulla oblongata; OL, occipital lobe; p, pons; TL, temporal lobe.

The female patient, of Roma ethnicity, was born from the parental second pregnancy. Parents were reportedly nonconsanguineous. Family history was unremarkable, except that the mother and maternal aunt were short‐statured. Delivery occurred at 39 weeks of gestation following induction with oxytocin. The birth weight was 2830 g (18th percentile, SD −0.97), length 46 cm (5th percentile, SD −1.69), and head circumference 31.1 cm (1st percentile, SD −2.25). The Apgar scores were 9 and 9 at 1st and 5th minute, respectively. Microcephaly was observed, with a disproportionately small neurocranium compared with the viscerocranium. On examination, the newborn exhibited scarce spontaneous movements, hypertonia, tremor, and opisthotonus. During the first day of life, the newborn developed respiratory insufficiency with hypothermia and required oxygen therapy for respiratory support over a 2‐day period. Phenobarbital was administered to manage tremor and irritability, and feeding was given via nasogastric tube. Brain MRI revealed reduced brain volume, simplification of the gyral pattern in both frontal lobes, and thin corpus callosum. The medulla oblongata was gracile, with more pronounced thinning of the pons (Figure 1A,B). Myelination was delayed in the perirolandic region, corona radiata, and dorsal pons. The ventricular system was normally positioned and sized, without evidence of hydrocephalus (Figure 1C,D). EEG demonstrated bilateral epileptiform abnormalities. Ophthalmological evaluation showed thin retinal pigment epithelium, while cardiac and abdominal ultrasound findings were normal.

Routine biochemical tests, plasma acylcarnitine profile, serum sialotransferrin analysis, urinary organic acids, and array comparative genomic hybridization showed no abnormalities. Plasma amino acid analysis of a sample collected 3 h after feeding showed asparagine concentration at the lower end of the reference range (30 μmol/L, ref. range 30–132). Aspartic acid (5 μmol/L, ref. range 0–20), glutamine (625 μmol/L, ref. range 370–958), and glutamic acid (49 μmol/L, ref. range 30–110) were within their reference ranges, as well as other amino acids in plasma, except for mildly decreased glycine (187 μmol/L, ref. range 224–514). However, CSF amino acid analysis revealed lowered asparagine (4.3 μmol/L, ref. range 5.9–11.2). Aspartic acid (0.6 μmol/L, ref. range 0–3.5), glutamine (552.4 μmol/L, ref. range 417.2–701.8), and glutamic acid (0.5 μmol/L, ref. range 0–0.9) in CSF were within their reference range, as well as other amino acids, except for mildly decreased valine (17 μmol/L, ref. range 18.2–25). The diagnosis of ASNSD was established by whole exome sequencing that revealed a homozygous likely pathogenic variant rs1363014007 (c.904‐1G>A) in the *ASNS* gene (NM_001673.5). The variant affects the splice acceptor site in intron 7, and in silico analyses (SNV Rf, SNV Ada, SpliceAI) predict that this variant is likely to have a damaging effect on protein structure and function [18, 19]. The variant is present in the GnomAD population database (rs1363014007, GnomAD NFE = 0.0009%).

In the following months, the patient exhibited arrested psychomotor development, worsening spasticity, absence of voluntary movements, lack of visual contact, and persistent epileptiform activity on EEG despite antiepileptic treatment. She succumbed to respiratory failure at the age of 6 months.

### 3.1 Postmortem Brain Tissue Analysis

Macroscopic examination of the brain showed symmetrical brain hemispheres with a normal pattern of primary and secondary gyri, and a simplified pattern of tertiary gyri. The brain was significantly smaller in size than expected (343 g; the expected weight of a 6‐month‐old infant is between 550 and 900 g) [20]. No intracranial hemorrhage or vascular abnormalities were observed.

**FIGURE 2:**
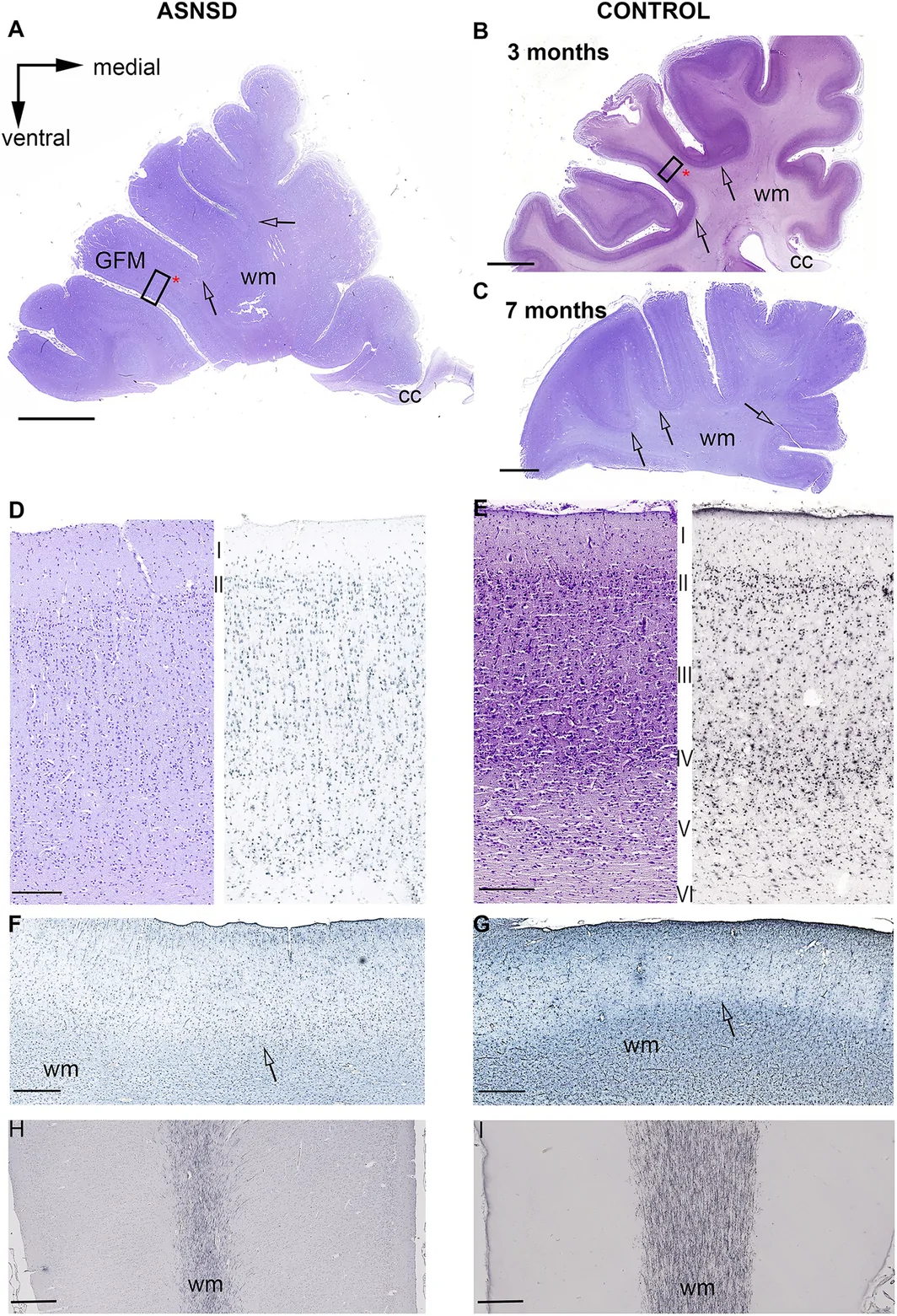
Disruption of the neuron and astrocyte arrangement in the prefrontal lobe of an infant with ASNSD compared with controls. The top images represent an overview of the prefrontal areas of the 6‐month old ASNSD patient (A), a 3‐month‐old (B), and a 7‐month‐old (C) infant, stained with Nissl staining. The areas marked with rectangles (in A and B) are shown enlarged in D and E, with Nissl staining (left), and immunohistochemically for NeuN (right), a pan‐neuronal marker. Note the sharp WM–cortex interface in both 3‐ and 7‐month‐old control brains (arrows in B, C, and G), while the ASNSD brain has a vague boundary (arrows in A and F), caused by increased cellularity of the WM. Cortical layers, although not definitive, are discernible already at 3 months (E), while cells in layers below Layer II in ASNSD (D) are disorganized. Astrocytes are visualized in (F, ASNSD patient) and (G, 3‐month‐old control brain) with immunoreactivity for glial fibrillary acidic protein (GFAP). In ASNSD (F), astrocytes are present in the cortex denser than in the control brain (G). Myelin‐basic protein immunoreactivity delineates myelinated fibers, thin and sparse in the 6‐month ASNSD patient (H) in comparison to the 3‐month‐old control brain (I). Areas shown in (H) and (I) were marked in (A) and (B) by a red asterisk. CC, corpus callosum; GFM, gyrus frontalis medius; WM, white matter. Scale bars: 5 mm in (A–C), 200 μm in (D and E), and 500 μm in (F–I).

Histological analysis revealed a disturbed arrangement of the gray and white matter (WM) in the telencephalon, causing reduced differentiation between them (Figure 2A–C,F,G). This is attributed to an increase in the overall cellular density of the WM. Prefrontal cortex in the ASNSD patient's brain (Figure 2D) was thinner than expected for age and comprised of a sharply bordered Layer I, vaguely bordered Layer II, while the underlying cortex was disrupted, without the expected laminar cortical architecture (laminar organization of the cortex is normally seen already at 3 months, Figure 2E right), and a dominantly radial rather than tangential neuronal orientation (Figure 2D right).

The other significant cellular population of the developing telencephalon is astrocytes, identified by immunoreactivity to glial fibrillary acidic protein (GFAP) (Figure 2F,G). During normal brain development, at the age of 3 months, the astrocytes have reached their final point, showing a mature‐like histoarchitectonical pattern: they are densely packed in the immature WM and markedly more sparse in the cortex (visible at 3 months in Figure 2G), which makes the cortex–WM interface easy to locate (arrow in Figure 2G). In the patient's brain, this border is indistinct (arrow in Figure 2F), due to a less pronounced difference in cell density between the cortex and WM. The myelination abnormality, previously described in other brain areas in MRI, is demonstrated immunohistochemically by myelin‐basic protein (MBP) immunoreactivity (Figure 2H,I).

**FIGURE 3:**
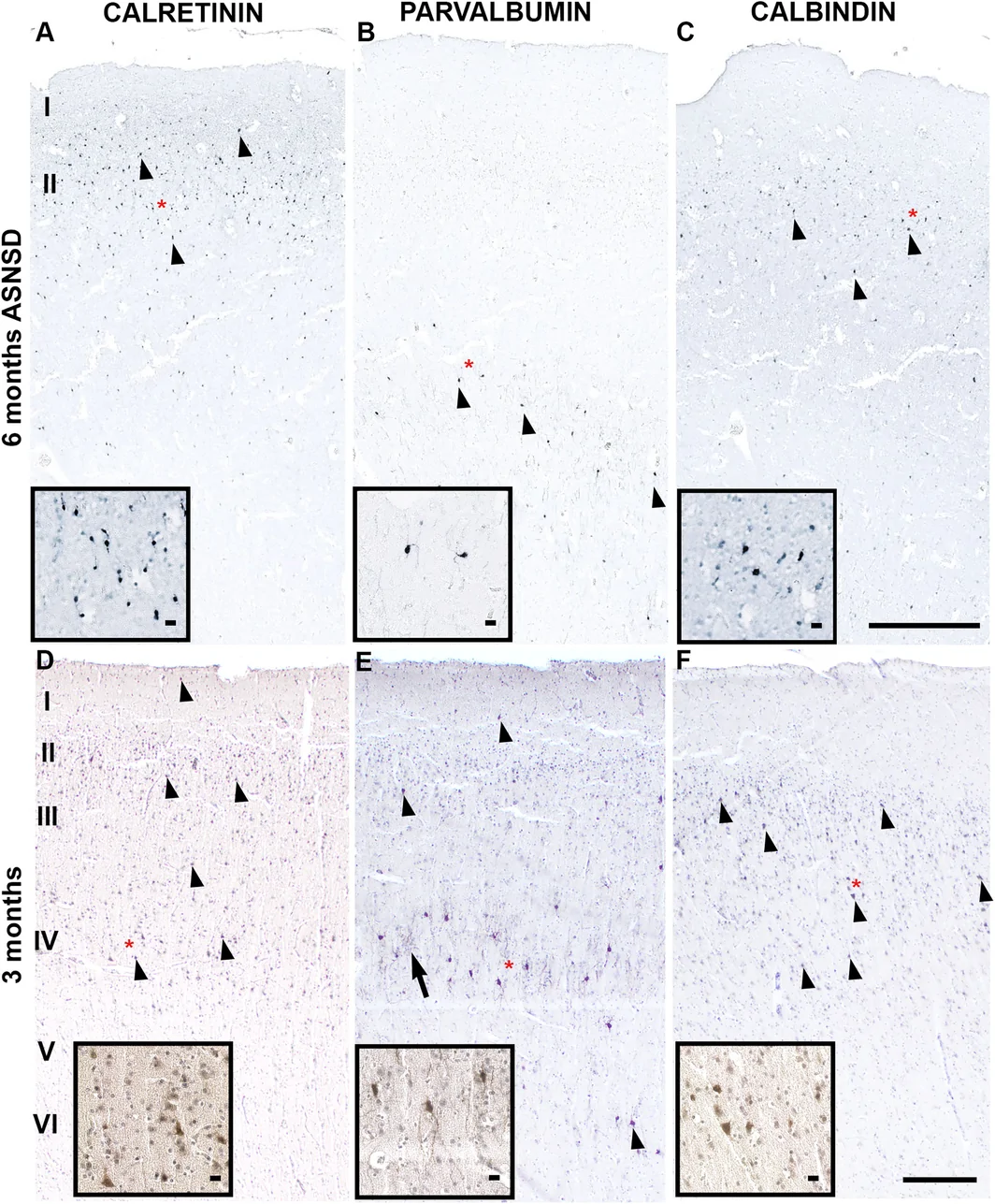
Density of calretinin (A, D), parvalbumin (B, E), and calbindin (C, F) immunoreactive interneurons in the cortex of the cingulate sulcus in the patient's (first row) and in the 3‐month‐old control brain (second row). The arrowheads mark interneuronal somas, which are less developed in size (most prominently seen in the case of parvalbumin) and number in ASNSD (A–C) in comparison to the control brain (D–F). The neuropil of parvalbumin neurons is underdeveloped in ASNSD compared with the control brain (*arrow* in E). The areas in the lower‐left corner of each histological section image show a higher‐magnification view of the region marked with a red asterisk, allowing the morphological details of the interneurons and neuropil to be visualized more clearly. Scale bars: 500 μm in (C), 200 μm in (F), 20 μm in all lower‐left corner higher‐magnification images.

The arrangement of interneuronal subpopulations further reflects the cortical disorganization in the patient's brain (Figure 3, first row). In the sulcus of the cingulate gyrus, parvalbumin (PARV)‐immunoreactive (ir) neurons were sparse, with an underdeveloped soma (Figure 3B, arrowheads) and a sparse neuropil in comparison to the control 3‐month‐old infant brain (Figure 3E). PARV‐ir interneurons are absent in the supragranular layers (II and III), where they would normally be found after reorganizational migration (Figure 3B). Calretinin (CALR)‐ir neurons (Figure 3A,D, arrowheads) are predominantly situated in the supragranular layers of the patient's brain, exhibiting a more mature distribution pattern than in the 3‐month‐old control brain (Figure 3D) where the CALR‐ir neurons are densely present both in supragranular layers and the temporary Layer IV (which will eventually spread its neurons to Layers III and V and form agranular cortex). Consequently, a less severe disruption of CALR‐ir interneurons' arrangement is visible than in the case of PARV‐ir neurons. The calbindin (CALB)‐ir neurons are situated in Layers II and III of the ASNSD brain (arrowheads in Figure 3C,F), reflecting an arrangement expected for the age and area, but less dense than expected.

While the basal ganglia and thalamus were histoarchitectonically developed as expected for the age, the claustrum was underdeveloped and consisted of a single‐cell layer (not shown).

In summary, the most striking histoarchitectonic findings in the telencephalon were individual neurons that arrested along their migratory path to the final destination in the gray matter, suggesting a widespread neuronal migration disorder that affects all analyzed neuronal subpopulations as well as astrocytes, resulting in a thin cortex with disorganized cortical layers.

## 4 Discussion

In this report, we present an infant diagnosed with ASNSD, with a comprehensive description of the clinical course, diagnostic process, and postmortem neuropathological findings of the brain. To the best of our knowledge, this is the first neuropathological description of the brain from an individual affected by ASNSD. The clinical presentation of our patient is consistent with the phenotypes described previously in the literature, as well as the brain MRI findings [2, 6]. Intracranial hemorrhage has been reported in two unrelated patients with ASNSD, but was not observed in our patient [21, 22]. Whether intracranial hemorrhage represents a direct manifestation of ASNSD or an infrequent associated finding remains uncertain.

Biochemical hallmarks of amino acid synthesis defects are more pronounced in CSF, as amino acid concentrations are not influenced by the absorption of amino acids from the diet [23]. Asparagine synthetase performs a transamidation reaction in which it converts aspartate to asparagine (amidation) with converting glutamine to glutamate (deamidation) [24]. Among the four amino acids involved in this reaction, asparagine is affected most prominently, because the concentrations of the other three amino acids can be maintained through alternative enzymes and metabolic pathways [25, 26]. Nevertheless, normal amino acid profiles both in plasma and CSF do not exclude ASNSD or other amino acid synthesis defects; therefore, the most reliable diagnostic method is genetic testing [10].

Although considered nonessential in humans, asparagine may become locally indispensable for proper brain development, as illustrated by the phenotype of patients with ASNSD and other inherited disorders affecting the biosynthesis of nonessential amino acids [6, 27, 28, 29, 30]. This dependence is likely explained by the limited and bidirectional transport of L‐asparagine across the blood–brain barrier, rendering cerebral asparagine concentrations largely dependent on synthesis within the central nervous system (CNS) [31, 32].

The highest levels of ASNS mRNA in the CNS are detected in the cerebral cortex, cerebellum, and hippocampus, as reported by the Human Protein Atlas [33]. In rodent neurodevelopmental studies, ASNS mRNA is expressed in the progenitor zones and cortical plate, the ultimate destination of migrating neurons, resembling the expression pattern of other known microcephaly‐causing genes [6, 24, 34, 35]. The mechanisms of abnormal brain development in ASNSD are not fully understood, but likely involve key developmental processes, including cell proliferation, neuronal migration, and myelination. Impaired cell proliferation and neuronal migration may underlie congenital microcephaly, whereas impaired myelination may contribute to the progressive postnatal neurological deterioration [36, 37]. Functional studies demonstrated that ASNSD patient‐derived fibroblasts failed to proliferate in asparagine‐depleted media, indicating dependence on endogenous asparagine synthesis when extracellular availability is limited [11]. In Asns‐deficient mice, cortical thinning, reduced brain surface area, and ventricular enlargement were observed [6]. Together, these experimental findings suggest that impaired endogenous asparagine synthesis disrupts corticogenesis. However, direct neuropathological evidence in humans has been lacking. In our patient, neuropathological examination revealed reduced neuronal density within the cerebral cortex and increased neuronal density in the underlying WM, abnormal cortical lamination, and radial neuronal orientation, consistent with neuronal migration and cortical orientation defect. These abnormalities disrupt normal microcircuit formation and may contribute to epileptogenesis, as supported by the abnormal distribution of interneurons, notably the near‐complete absence of parvalbumin‐positive interneurons in the supragranular layers [38, 39, 40, 41, 42].

Several molecular pathways may underlie these abnormalities. There is increasing evidence that asparagine has a regulatory role in intracellular signaling pathways involved in cell proliferation and survival. It has been recognized as an activator of the mTORC1 signaling pathway, both directly and indirectly, thereby promoting cell growth and proliferation [43, 44]. The mTOR pathway plays a central role in brain development, and its dysregulation has been implicated in abnormal neuronal development and structural brain abnormalities [45]. Furthermore, asparagine modulates the activity of LKB1 kinase, which regulates neuronal polarization and axon initiation [46, 47]. In developing cortical neurons in vivo, both downregulation and overexpression of LKB1 abolished axon formation [47]. Since asparagine has a regulatory role in LKB1 signaling, its deficiency may impair axon differentiation. Together, these mechanisms may contribute to the structural brain malformations observed in ASNSD.

Oral L‐asparagine supplementation has been attempted as a therapeutic approach in ASNSD. In the first reported patient, worsening of epilepsy was observed; however, a study of two siblings suggested a potentially beneficial effect in stabilizing the disease course and preventing further developmental regression, without seizure exacerbation [48, 49]. A possible limitation of this approach is the reduced transport of asparagine across the blood–brain barrier, as reflected by its lower concentration in CSF than in plasma [23]. Late diagnostic confirmation precluded a trial of L‐asparagine supplementation in our patient. Another hypothetical therapeutic strategy is glutamine supplementation. Because glutamine readily crosses the blood–brain barrier and serves as the amino group donor for ASNS, increasing its intracellular availability could theoretically enhance residual ASNS‐mediated asparagine synthesis [50]. To our knowledge, the therapeutic potential of glutamine supplementation has not been evaluated in ASNSD. A major challenge for all therapeutic strategies is that abnormal brain development in ASNSD begins during fetal life, which limits the efficacy of postnatal interventions. Our neuropathological findings of disrupted cortical organization and neuronal migration further support the need for prenatal therapeutic strategies.

## 5 Conclusion

This report presents the first neuropathological characterization of a patient with ASNSD. Histological analysis revealed increased neuronal density in the WM and abnormal cortical lamination, consistent with profound disruption of neuronal migration. These findings highlight the critical role of asparagine in cortical development and suggest that reduced intracellular asparagine availability may impair neural progenitor cell proliferation, leading to abnormal cortical development and microcephaly. Although limited to a single case, this study provides valuable insight into the neuropathological features of ASNSD and establishes a foundation for future studies on the pathogenesis of this rare neurometabolic disorder.

## Author Contributions

Danijela Petković Ramadža, Tamara Žigman, Ivo Barić, Sanda Huljev Frković, Viktorija Antolović, Bernarda Medlobi Vinković, Nenad Kešin, and Ivan Lehman were involved in patient management. Mirjana Novoselec established the neuroradiological diagnosis. Josipa Mateševac performed NGS analysis. Ana Škaričić performed biochemical analysis. Monica Kirigin Kalamar performed autopsy; Mihaela Bobić, Goran Sedmak, Ema Bokulić, and Tila Medenica analyzed postmortem brain tissue. Ema Bokulić and Tila Medenica did the histological and immunohistochemical part of the study. Mihaela Bobić, Goran Sedmak, and Danijela Petković Ramadža wrote the first draft. Conceptualization was done by Goran Sedmak and Danijela Petković Ramadža. All authors critically reviewed the manuscript.

## Funding

This publication was co‐financed by the Scientific Centre of Excellence for Basic, Clinical and Translational Neuroscience (project “Development of protocol for early diagnosis of hypoxic–ischemic brain lesion in infancy and early childhood”; GA PK1.1.10.0009 funded by the European Union through the European Regional Development Fund).

## Ethics Statement

The use of human postmortem brain tissue was approved by the Ethics Committee of the University of Zagreb School of Medicine (approval number 251‐59‐10106‐23‐111/152).

## Consent

Written informed consent for publication of this case report and the accompanying images was obtained from the patient's mother, acting as next of kin.

## Conflicts of Interest

The authors declare no conflicts of interest.

## Data Availability

The data and materials supporting the findings of this study are available from the authors upon reasonable request.

## References

[1] M. Alfadhel and A. W. El‐Hattab , “Asparagine Synthetase Deficiency,” accessed May7, 2026, https://www.ncbi.nlm.nih.gov/books/NBK525916. 30234940

[2] M. Alfadhel , M. T. Alrifai , D. Trujillano , et al., “Asparagine Synthetase Deficiency: New Inborn Errors of Metabolism,” JIMD Reports 22 (2015): 11–16. 25663424 10.1007/8904_2014_405 PMC4486270

[3] M. C. Chang , S. J. Staklinski , M. E. Merritt , and M. S. Kilberg , “A Method for Measurement of Human Asparagine Synthetase (ASNS) Activity and Application to ASNS Protein Variants Associated With ASNS Deficiency,” Biological Methods and Protocols 8, no. 1 (2023): bpad026. 10.1093/biomethods/bpad026 PMC10641120 37965492

[4] E. Alharby , E. A. Faqeih , M. Saleh , et al., “Clinical, Molecular, and Biochemical Delineation of Asparagine Synthetase Deficiency in Saudi Cohort,” Genetics in Medicine 22, no. 12 (2020): 2071–2080. 32741967 10.1038/s41436-020-0919-x

[5] C. Galada , M. Hebbar , L. Lewis , et al., “Report of Four Novel Variants in ASNS Causing Asparagine Synthetase Deficiency and Review of Literature,” Congenital Anomalies 58, no. 5 (2018): 181–182. 29405484 10.1111/cga.12275 PMC6338226

[6] E. K. Ruzzo , J. M. Capo‐Chichi , B. Ben‐Zeev , et al., “Deficiency of Asparagine Synthetase Causes Congenital Microcephaly and a Progressive Form of Encephalopathy,” Neuron 80, no. 2 (2013): 429–441. 24139043 10.1016/j.neuron.2013.08.013 PMC3820368

[7] N. Gupta , V. V. Tewari , M. Kumar , et al., “Asparagine Synthetase Deficiency‐Report of a Novel Mutation and Review of Literature,” Metabolic Brain Disease 32, no. 6 (2017): 1889–1900. 28776279 10.1007/s11011-017-0073-6

[8] J. Sun , A. J. McGillivray , J. Pinner , et al., “Diaphragmatic Eventration in Sisters With Asparagine Synthetase Deficiency: A Novel Homozygous ASNS Mutation and Expanded Phenotype,” JIMD Reports 34 (2016): 1–9. 27469131 10.1007/8904_2016_3 PMC5509547

[9] T. Yamamoto , W. Endo , H. Ohnishi , et al., “The First Report of Japanese Patients With Asparagine Synthetase Deficiency,” Brain & Development 39, no. 3 (2017): 236–242. 27743885 10.1016/j.braindev.2016.09.010

[10] T. J. De Koning , “Amino Acid Synthesis Deficiencies,” Journal of Inherited Metabolic Disease 40, no. 4 (2017): 609–620, 10.1007/s10545-017-0063-1. 28653176 PMC5500668

[11] E. E. Palmer , J. Hayner , R. Sachdev , et al., “Asparagine Synthetase Deficiency Causes Reduced Proliferation of Cells Under Conditions of Limited Asparagine,” Molecular Genetics and Metabolism 116, no. 3 (2015): 178–186. 26318253 10.1016/j.ymgme.2015.08.007 PMC10152381

[12] S. Ben‐Salem , J. G. Gleeson , A. M. Al‐Shamsi , et al., “Asparagine Synthetase Deficiency Detected by Whole Exome Sequencing Causes Congenital Microcephaly, Epileptic Encephalopathy and Psychomotor Delay,” Metabolic Brain Disease 30, no. 3 (2015): 687–694. 25227173 10.1007/s11011-014-9618-0 PMC4915861

[13] M. Z. Seidahmed , M. A. Salih , O. B. Abdulbasit , et al., “Hyperekplexia, Microcephaly and Simplified Gyral Pattern Caused by Novel ASNS Mutations, Case Report,” BMC Neurology 16 (2016): 105. 27422383 10.1186/s12883-016-0633-0 PMC4947274

[14] D. Schleinitz , A. Seidel , R. Stassart , et al., “Novel Mutations in the Asparagine Synthetase Gene (ASNS) Associated With Microcephaly,” Frontiers in Genetics 13, no. 9 (2018): 245. 10.3389/fgene.2018.00245 PMC6053511 30057589

[15] S. J. Staklinski , M. C. Chang , F. Yu , et al., “Cellular and Molecular Characterization of Two Novel Asparagine Synthetase Gene Mutations Linked to Asparagine Synthetase Deficiency,” Journal of Biological Chemistry 298, no. 9 (2022): 102385. 35985424 10.1016/j.jbc.2022.102385 PMC9478401

[16] C. Wang , G. He , Y. Ge , R. Li , Z. Li , and Y. Lin , “A Novel Compound Heterozygous Missense Mutation in ASNS Broadens the Spectrum of Asparagine Synthetase Deficiency,” Molecular Genetics & Genomic Medicine 8, no. 6 (2020): e1235. 32255274 10.1002/mgg3.1235 PMC7284041

[17] M. Judaš , G. Šimic , Z. Petanjek , et al., “The Zagreb Collection of Human Brains: A Unique, Versatile, but Underexploited Resource for the Neuroscience Community,” Annals of the New York Academy of Sciences 1225, no. Suppl 1 (2011): E105–E130. 21599691 10.1111/j.1749-6632.2011.05993.x

[18] X. Jian , E. Boerwinkle , and X. Liu , “In Silico Prediction of Splice‐Altering Single Nucleotide Variants in the Human Genome,” Nucleic Acids Research 42, no. 22 (2014): 13534–13544. 25416802 10.1093/nar/gku1206 PMC4267638

[19] K. Jaganathan , P. S. Kyriazopoulou , J. F. McRae , et al., “Predicting Splicing From Primary Sequence With Deep Learning,” Cell 176, no. 3 (2019): 535–548. 30661751 10.1016/j.cell.2018.12.015

[20] A. R. Bamber , S. M. L. Paine , D. A. Ridout , J. W. Pryce , T. S. Jacques , and N. J. Sebire , “Brain Weight in Sudden Unexpected Death in Infancy: Experience From a Large Single‐Centre Cohort,” Neuropathology and Applied Neurobiology 42, no. 4 (2016): 344–351. 26095474 10.1111/nan.12251

[21] A. Radha Rama Devi and S. M. Naushad , “Molecular Diagnosis of Asparagine Synthetase (ASNS) Deficiency in Two Indian Families and Literature Review of 29 ASNS Deficient Cases,” Gene 1, no. 704 (2019): 97–102. 10.1016/j.gene.2019.04.024 30978478

[22] G. M. H. Abdel‐Salam and M. S. Abdel‐Hamid , “Asparagine Synthetase Deficiency With Intracranial Hemorrhage Can Mimic Molybdenum Cofactor Deficiency,” Neuropediatrics 52, no. 3 (2021): 201–207. 33271615 10.1055/s-0040-1718917

[23] S. Scholl‐Bürgi , E. Haberlandt , P. Heinz‐Erian , et al., “Amino Acid Cerebrospinal Fluid/Plasma Ratios in Children: Influence of Age, Gender, and Antiepileptic Medication,” Pediatrics 121, no. 4 (2008): e920–e926. 18332074 10.1542/peds.2007-1631

[24] C. L. Lomelino , J. T. Andring , R. McKenna , and M. S. Kilberg , “Asparagine Synthetase: Function, Structure, and Role in Disease,” Journal of Biological Chemistry 292, no. 49 (2017): 19952–19958. 29084849 10.1074/jbc.R117.819060 PMC5723983

[25] C. D. Rae , B. D. Rowlands , and V. J. Balcar , “Aspartate in the Brain: A Review,” Neurochemical Research 50, no. 3 (2025): 199. 40506607 10.1007/s11064-025-04454-3 PMC12162812

[26] R. Dalangin , A. Kim , and R. E. Campbell , “The Role of Amino Acids in Neurotransmission and Fluorescent Tools for Their Detection,” International Journal of Molecular Sciences 21, no. 17 (2020): 6197. 32867295 10.3390/ijms21176197 PMC7503967

[27] M. R. Baumgartner , A. C. Hu , S. Almashanu , et al., “Hyperammonemia With Reduced Ornithine, Citrulline, Arginine and Proline: A New Inborn Error Caused by a Mutation in the Gene Encoding Delta1‐Pyrroline‐5‐Carboxylate Synthase,” Human Molecular Genetics 9, no. 19 (2000): 2853–2858. 11092761 10.1093/hmg/9.19.2853

[28] R. Acuna‐Hidalgo , D. Schanze , A. Kariminejad , et al., “Neu‐Laxova Syndrome Is a Heterogeneous Metabolic Disorder Caused by Defects in Enzymes of the L‐Serine Biosynthesis Pathway,” American Journal of Human Genetics 95, no. 3 (2014): 285–293. 25152457 10.1016/j.ajhg.2014.07.012 PMC4157144

[29] J. Häberle , B. Görg , F. Rutsch , et al., “Congenital Glutamine Deficiency With Glutamine Synthetase Mutations,” New England Journal of Medicine 353, no. 18 (2005): 1926–1933. 16267323 10.1056/NEJMoa050456

[30] A. Larkin and B. Imperiali , “The Expanding Horizons of Asparagine‐Linked Glycosylation,” Biochemistry 50, no. 21 (2011): 4411–4426. 21506607 10.1021/bi200346n PMC3101296

[31] R. Zaragozá , “Transport of Amino Acids Across the Blood‐Brain Barrier,” Frontiers in Physiology 23, no. 11 (2020): 973. 10.3389/fphys.2020.00973 PMC7538855 33071801

[32] K. M. Hargreaves and W. M. Pardridge , “Neutral Amino Acid Transport at the Human Blood‐Brain Barrier,” Journal of Biological Chemistry 263, no. 36 (1988): 19392–19397. 2848825

[33] M. Uhlén , L. Fagerberg , B. M. Hallström , et al., “Tissue‐Based Map of the Human Proteome,” Science 347, no. 6220 (2015): 1260419. 25613900 10.1126/science.1260419

[34] J. Bond , E. Roberts , G. H. Mochida , et al., “ASPM Is a Major Determinant of Cerebral Cortical Size,” Nature Genetics 32, no. 2 (2002): 316–320. 12355089 10.1038/ng995

[35] A. P. Jackson , H. Eastwood , S. M. Bell , et al., “Identification of Microcephalin, a Protein Implicated in Determining the Size of the Human Brain,” American Journal of Human Genetics 71, no. 1 (2002): 136–142. 12046007 10.1086/341283 PMC419993

[36] D. Alcantara and M. O'Driscoll , “Congenital Microcephaly,” American Journal of Medical Genetics—Seminars in Medical Genetics, Part C 166C, no. 2 (2014): 124–139. 10.1002/ajmg.c.31397 24816482

[37] S. Passemard , F. Perez , E. Colin‐Lemesre , S. Rasika , P. Gressens , and V. El Ghouzzi , “Golgi Trafficking Defects in Postnatal Microcephaly: The Evidence for Golgipathies,” Progress in Neurobiology 153 (2017): 46–63. 28377289 10.1016/j.pneurobio.2017.03.007

[38] R. Coras , H. Holthausen , and H. B. Sarnat , “Focal Cortical Dysplasia Type 1,” Brain Pathology 31, no. 4 (2021): e12964. 34196986 10.1111/bpa.12964 PMC8412088

[39] J. B. Ruden , L. L. Dugan , and C. Konradi , “Parvalbumin Interneuron Vulnerability and Brain Disorders,” Neuropsychopharmacology 46, no. 2 (2021): 279–287. 32722660 10.1038/s41386-020-0778-9 PMC7852528

[40] P. L. A. Gabbott , P. R. Jays , and S. J. Bacon , “Calretinin Neurons in Human Medial Prefrontal Cortex (Areas 24a,b,c, 32′, and 25),” Journal of Comparative Neurology 381, no. 4 (1997): 389–410. 9136798 10.1002/(sici)1096-9861(19970519)381:4<389::aid-cne1>3.0.co;2-z

[41] D. Sedmak , Calretinin Neurons in the Primate Prefrontal Cortex [Dissertation] (University of Zagreb, 2019), accessed May 7, 2026, https://urn.nsk.hr/urn:nbn:hr:105:991608.

[42] I. Banovac , D. Sedmak , M. Esclapez , and Z. Petanjek , “The Distinct Characteristics of Somatostatin Neurons in the Human Brain,” Molecular Neurobiology 59, no. 8 (2022): 4953–4965. 35665897 10.1007/s12035-022-02892-6

[43] D. Meng , Q. Yang , H. Wang , et al., “Glutamine and Asparagine Activate mTORC1 Independently of Rag GTPases,” Journal of Biological Chemistry 295, no. 10 (2020): 2890–2899. 32019866 10.1074/jbc.AC119.011578 PMC7062167

[44] M. Laplante and D. M. Sabatini , “mTOR Signaling at a Glance,” Journal of Cell Science 122, no. 20 (2009): 3589–3594. 19812304 10.1242/jcs.051011 PMC2758797

[45] N. Takei and H. Nawa , “mTOR Signaling and Its Roles in Normal and Abnormal Brain Development,” Frontiers in Molecular Neuroscience 23, no. 7 (2014): 28. 10.3389/fnmol.2014.00028 PMC4005960 24795562

[46] A. P. Barnes , B. N. Lilley , Y. A. Pan , et al., “LKB1 and SAD Kinases Define a Pathway Required for the Polarization of Cortical Neurons,” Cell 129, no. 3 (2007): 549–563. 17482548 10.1016/j.cell.2007.03.025

[47] M. Shelly , L. Cancedda , S. Heilshorn , G. Sumbre , and M. M. Poo , “LKB1/STRAD Promotes Axon Initiation During Neuronal Polarization,” Cell 129, no. 3 (2007): 565–577. 17482549 10.1016/j.cell.2007.04.012

[48] M. T. Alrifai and M. Alfadhel , “Worsening of Seizures After Asparagine Supplementation in a Child With Asparagine Synthetase Deficiency,” Pediatric Neurology 58 (2016): 98–100. 27268761 10.1016/j.pediatrneurol.2016.01.024

[49] R. Sprute , D. Ardicli , K. K. Oguz , et al., “Clinical Outcomes of Two Patients With a Novel Pathogenic Variant in ASNS: Response to Asparagine Supplementation and Review of the Literature,” Human Genome Variation 6, no. 1 (2019): 24. 31123592 10.1038/s41439-019-0055-9 PMC6531480

[50] B. Olawuni and B. P. Bode , “Asparagine as a Signal for Glutamine Sufficiency via Asparagine Synthetase: A Fresh Evidence‐Based Framework in Physiology and Oncology,” American Journal of Physiology. Cell Physiology 327, no. 5 (2024): C1335–C1346. 39344414 10.1152/ajpcell.00316.2024

